# Using psychometric and focus groups methods to assess patients' attitudes regarding the role of dental providers and vaccinations for COVID-19 and HPV

**DOI:** 10.3389/froh.2026.1740318

**Published:** 2026-02-20

**Authors:** Tamara J. Cadet, Tyler M. Moore, Yueping Luo, Morgan Faist, Chelsea K. Brown, Jinbo Niu, Daphne Hicyilmaz, Katherine France

**Affiliations:** 1 University of Pennsylvania School of Social Policy & Practice, Philadelphia, PA, United States; 2 Division of Community Oral Health, University of Pennsylvania School of Dental Medicine, Philadelphia, PA, United States; 3 Department of Penn Psychiatry, University of Pennsylvania Perelman School of Medicine, Philadelphia, PA, United States; 4 University of Pennsylvania School of Arts & Sciences, Philadelphia, PA, United States; 5 University of Southern California, Los Angeles, CA, United States; 6 Department of Oral Medicine, University of Pennsylvania School of Dental Medicine, Philadelphia, PA, United States

**Keywords:** dentists, focus group, health promotion, HPV, psychometric, survey, vaccine COVID-19

## Abstract

**Objectives:**

To develop a initial validated survey for assessment of patient perceptions around dental provider role in HPV and COVID-19 vaccination.

**Methods:**

Using a parallel convergent mixed methods design, this study reports the initial validation of a survey through the integration of psychometric and focus group methods and presents findings of patient perceptions of dentist involvement in vaccine efforts through a focus group in Philadelphia, PA.

**Results:**

A previously tested pilot survey was modified based on the integrated results. The final survey was organized according to factor analysis and reflected the focus group recommendations. The changes reduced the survey by 25% from 22 to 16 questions. Two overarching themes to better understand perceptions of dentist involvement in vaccine efforts identified from the focus group were: 1. attitudes related to appropriateness and acceptability of dental providers educating and administering vaccinations; and 2. how lack of knowledge hindered intentions to seek vaccination from dental providers.

**Conclusion:**

The combination of the psychometric and focus group analyses resulted in a final survey to assess patient's attitudes and acceptance of HPV and COVID-19 vaccination, including vaccination status, knowledge about the impacts of HPV and COVID-19, perceptions of dental care providers’ role, and comfort in having a dental provider administer HPV and COVID-19 vaccines.

**Practice and policy implications:**

This initial validity process of the survey from creates an opportunity to assess patient acceptance of dentist roles in vaccine efforts around HPV and COVID-19. Further validation of the final form is pending.

## Introduction

Vaccinations have long been a central tool of public health (1). Two illnesses for which vaccines are widely available include COVID-19 and Human papillomavirus (HPV). COVID-19 caused widespread global illness and death (2). In response to the unprecedented impacts of COVID-19 on individual and societal health and wellbeing, vaccines against the virus were developed with historic speed. The three vaccines against COVID-19 currently authorized in the United States are manufactured by the companies Pfizer-BioNTech, Moderna, and Novavax (3). During the COVID-19 pandemic, restrictions intended to limit the spread of the disease also resulted in decreased national vaccine uptake and rates of complete vaccination, including against HPV (4).

HPV is the most common sexually transmitted infection in the United States. Most HPV infections are cleared without manifestation or secondary impact. However, some can cause genital and oral warts and can lead to certain types of cancers including cervical, vaginal, penile, anal and up to 70% of throat cancers (5). Vaccines against HPV were first approved for use in 2006 and prevent more than 90% of HPV-related cancers. HPV vaccination is recommended for everyone through age 26. Catch up vaccination is provided for some adults between 27 and 45 years of age (6).

Vaccination is a highly effective intervention for the prevention of HPV infection and its secondary effects and prevention of serious disease and death due to COVID-19 (3, 7). Multiple states include dentists and dental health professionals in the community of providers authorized to deliver both COVID-19 and HPV vaccines (8, 9). Previous research examined patient and dental provider attitudes around the provision of vaccines in the dental setting (10, 11). Much of this research focused on the HPV vaccine series given the increasing rates of HPV-positive oropharyngeal cancers (12–14). However, no previous research has developed or deployed a validated survey instrument for assessment of these perceptions. To address this gap, a prior study (1) developed a questionnaire based on previously published research to assess patient perceptions of dentist involvement in vaccine efforts.

The primary aim of the current study was to conduct an initial validation of this questionnaire using a mixed methods approach. A secondary aim was to further understand perceptions of dentist involvement in vaccine efforts. Initial validation of the survey instrument will ensure the accuracy and legitimacy of additional survey results along with the applicability of data obtained. Institutional Review Board approval was received for this study.

## Methods

Using a parallel convergent mixed methods design, this study reports the findings from the initial validation of our survey through psychometric and focus group methods (15). Combining both quantitative and qualitative methods can offer additional insights into the quality of the questionnaire and provide more rigorous validity evidence (16, 17). Further, this design examines patient perceptions around feasibility and acceptability of dental provider involvement in vaccinations.

### Pilot questionnaire

The questionnaire used as part of a Steinbaum et al., (2023) study consisted of 22 items and two open-ended questions adapted from a previously published survey [herein referred to as pilot survey] on parent acceptance of dentist roles in HPV vaccination (Supplementary Appendix A) (18). Of the 22 items, 6 were demographic questions; 8 were related to COVID-19 vaccinations and 8 were related to the HPV vaccination. Questions between the COVID-19 and HPV vaccinations were similar in content assessing whether participants were up to date in their vaccinations, their knowledge of the vaccines and their beliefs and comfort with dental professionals administering vaccines. For all analyses below, responses of “unsure” or “prefer not to answer” were coded as missing, making all responses binary (yes/no).

### Quantitative study

The quantitative study aimed to evaluate the psychometric properties of the pilot questionnaire, including construct validity assessment using exploratory factor analysis (EFA) and computerized adaptive test (CAT) simulation (19). We used the data from the 163 participants in the Steinbaum et al., (2023) study to calibrate the items. The senior author on the current paper is a co-author on the Steinbaum et al. (2023) paper providing access to the data for 163 participants The data for these participants were used to conduct the psychometric analyses. For the 163 participants in the Steinbaum et al., (2023) study, patients ages 18–45 with dental appointments and no limitations according to medical condition, race, other demographic factors, or dental history were recruited from an academic dental institution in Pennsylvania using a convenience sampling method.

**Figure 1:**
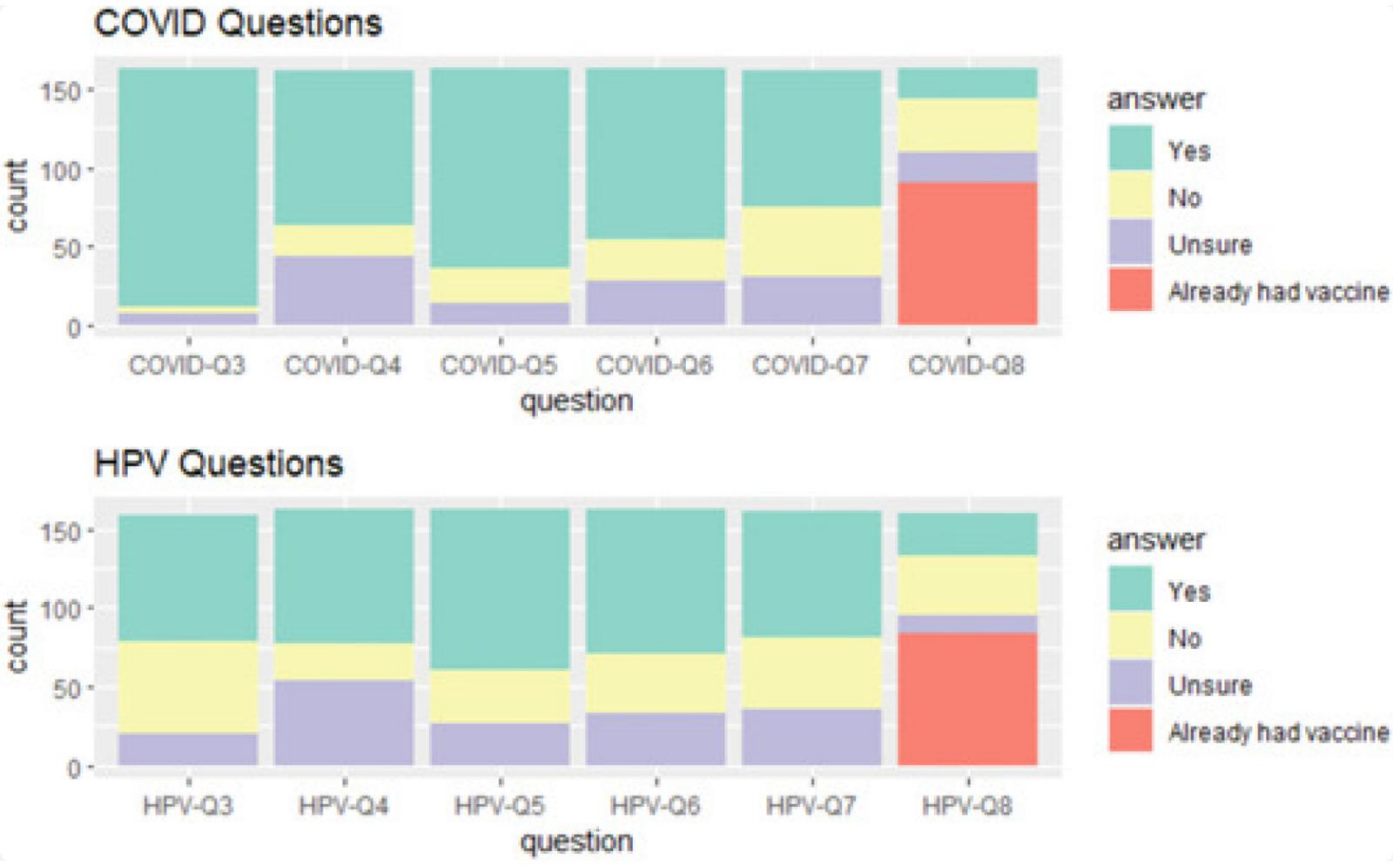
Pilot survey distribution. Graphic representation survey response distribution by question, divided into COVID-19 and HPV categories. “COVID-Q3” refers to whether respondents were aware that COVID-19 vaccines can prevent severe illness caused by COVID-19. “HPV-Q3” refers to whether respondents were aware that HPV vaccines can prevent some forms of head and neck cancers. “COVID-Q4” and “HPV-Q4” refer to whether respondents believed dental providers are qualified to educate about each vaccine. “COVID-Q5” and “HPV-Q5” refer to whether respondents feel comfortable discussing each vaccine with their dental provider. “COVID-Q6” and “HPV-Q6” refer to whether respondents would accept a recommendation for each vaccine from their dental provider. “COVID-Q7” and “HPV-Q7” refer to whether respondents would feel comfortable allowing a dental provider to administer each vaccine. “COVID-Q8” and “HPV-Q8” refer to whether respondents would accept each vaccine if offered today. Reproduced from Steinbaum S, Jagannath J, Seymour L, Corby P, Kulkarni R, France K. Oral healthcare providers play a vital role in vaccination efforts: patient perspectives. *Clin Exp Dent Res*. (2023) 9(6):1169–79. doi: 10.1002/cre2.777 licensed under CC BY 4.0.

Participants were primarily female (67%) with a mean age of 31.67 (7.67). Forty-four percent of participants identified as African American/Black followed by Whites (32%). Slightly more than half had less than a high school degree (26%) or some college (27%) with one-third having an income less than $20,000 and one-third between $35,000 and $75,000. About one-half of participants were vaccinated against each of COVID-19 (55%) and HPV (51%). See Figure 1 for pilot survey distribution results. Data were collected on sex assigned at birth, gender identity, age, race, education, household income, vaccination status and plans, education about the vaccines, comfort, and attitudes about the dental provider's role in vaccinations for both COVID-19 and HPV. For analysis, age and race was simplified into 3-level categorical variables (younger than 24, ages 24–40, and 40 and older and White, African American/Black, and others, respectively). Education level was simplified into less than high school, some college, bachelor's degree, and graduate degrees. When analyzing responses for whether patients would be comfortable receiving a vaccine today (HPV or COVID-19), individuals already vaccinated were removed from analysis of this question only. Other than noting that the percentages did not add to 100 due to missingness, no further information was provided. See Table 1 for data regarding missing questions.

**Table 1:** Number of missing responses to questions.

1 missing response	2 missing responses	3 missing responses	4 missing responses
1. Do you believe dental professionals are qualified to educate you about the COVID-19 vaccines available? 2. Would you feel comfortable allowing a dental provider to administer a COVID-19 vaccine for you? 3. Do you believe dental professionals are qualified to educate you about the human papillomavirus (HPV) vaccines available? 4. Would you feel comfortable discussing human papillomavirus (HPV) vaccines with your dental provider? 5. Would you accept the recommendation for a human papillomavirus (HPV) vaccine from your dental provider? 6. 6. If your dental provider offered you a human papillomavirus (HPV) vaccine today, you would likely:	1. If your dental provider offered you a COVID-19 vaccine today, you would likely: 2. Would you feel comfortable allowing a dental provider to administer a human papillomavirus (HPV) vaccine for you?	Have you been completely vaccinated against human papillomavirus (HPV)?	Have you been informed that the human papillomavirus (HPV) vaccine can prevent some types of head and neck cancer?

Details of the CAT simulation method of test abbreviation are available (20). Briefly, CAT in its full implementation is a method of “catering” a test or scale to an examinee by continually updating (estimating and re-estimating) his/her trait level during the administration to determine the optimal next item (question) to administer (21, 22). A secondary use of CAT, when it is not desirable or feasible to develop a full CAT implementation system, is to simulate what would happen if a particular fixed-length scale (as here) were administered adaptively. The benefit of these simulations is that they reveal which items within a scale are most informative overall; for example, if there is an item that is never (or very rarely) administered in the simulations, one can infer that said item is “low quality” and could possibly be removed from the scale without noticeable loss of information. This approach to test and scale abbreviation has been used successfully in both cognitive (23, 24) and non-cognitive (25, 26) domains.

### Qualitative study

The qualitative study aimed to determine the clarity and accessibility of the pilot survey and to gain a deeper understanding of the perceptions of dental administration of vaccines through a focus group discussion (27). Focus groups are increasingly used in dental studies seeking a depth of understanding not captured in quantitative approaches (28, 29). The use of a focus group instead of individual interviews was selected so participants could provide their views and learn how their views were shaped by the synergy of the focus group (30). Focus groups allow for times when an individual participant may not have an immediate response but end up having one based on the conversation, which can produce rich, detailed data (31).

### Qualitative data collection

Using convenience sampling, we recruited 9 participants for a focus group using flyers distributed within Philadelphia, PA and online using a waiting room television screen where the recruitment flyer was advertised. Our target population was adults ages 18–45 with dental appointments. There were no limitations according to medical condition, race, other demographic factors, or dental history. Participants who met the following eligibility criteria were included: (1) patients between the ages of 18 and 45 to match possible eligibility for HPV vaccination; (2) ability to speak and read English; and (3) ability to independently consent to participate. A focus group facilitated by a trained moderator explores the perspectives of a group of participants about a particular problem—in this case—the clarity and accessibility of our pilot survey. Focus groups of 8–12 can provide information about the processes participants use to answer survey questions and assist researchers in learning how participants perceive items. In this case, 8 participants is considered an adequate size to facilitate discussion. Participants were asked to commit one hour to the review of a pilot survey and were offered a $50 gift card and a meal as incentives for participation.

Guided by the Health Belief Model (32), we gathered participants’ feedback on the questions in the pilot survey to understand if the questions were clear, how they thought about the questions, and their opinions about the questions. See Supplementary Table 2: Appendix B. All patients were consented prior to starting the focus group. The focus group was recorded for transcription and coding purposes. A moderator along with a lead and an assistant notetaker facilitated the focus group. Using the think-aloud method, the focus group discussion centered on comprehension, understanding, meaning of questions and satisfaction with the questionnaire (33). The think-aloud approach allows researchers to understand how patients perceive and interpret the information presented and to identify any potential problems in the material. Specifically, we reviewed the questionnaire in detail and asked participants to say everything that passed through their minds as they read. Participants were probed in any areas where there seemed to be confusion.

### Quantitative data analysis

For the psychometric analyses, we conducted exploratory item-factor analysis and computerized adaptive test (CAT) simulation (20). Item covariance structure was assessed using exploratory item-factor analysis (item-wise EFA). Inter-item tetrachoric correlations were calculated, and 1-, 2-, and 3-factor solutions were estimated using least-squares extraction and promax rotation. Four methods for determining the optimal number of factors were used: the minimum average partial (MAP) method (34), parallel analysis with Glorfeld correction at the 95th percentile (35), the minimum Bayesian Information Criterion (36), and subjective evaluation of the scree plot to determine where the successively decreasing eigenvalues begin to form a linear trend (the “elbow”). These methods suggested 1-factor, 1-factor, 3-factor, and 1-factor solutions, respectively. The 1-factor (unidimensional) result was supported by the ratio of first to second eigenvalues of 8.3/1.4 = 5.9, which is beyond a commonly used cutoff of 3.0 (23). EFA solutions from one to three factors were estimated for thoroughness. For the above factor analytic procedures, missing data were handled with pairwise deletion.

For theoretical reasons, and to suit the goals of the original protocol, it was necessary to keep the COVID-related items separate from the HPV-related items. The goal of the study was to compare attitudes between the different disease states (not knowledge, which we used to support attitudes and not behavior but rather intention) thus despite the EFA results that suggest a single factor. The second step was therefore to develop two abbreviated forms of the 8-item scales using computerized adaptive test (CAT) simulation (19). Items were calibrated in preparation for CAT-simulation using the 2-parameter logistic item response theory model (2PLM) using the *mirt* package in R, with missing data handled using full-information maximum likelihood (FIML) (37). Details of IRT are beyond the present scope (38), but the goal of the method is to characterize each item (question) using some quantifiable parameters. The 2PLM characterizes items in terms of their discrimination (α) and difficulty/extremity (β). The discrimination parameter indicates how precisely an item can distinguish among individuals on a particular trait continuum (here, negative attitude toward COVID or HPV vaccines), where highly discriminating items are generally desirable (high quality). The difficulty/extremity parameter indicates how “far up” the trait continuum someone needs to be to have a 50% probability of endorsing the item. An important feature of IRT models is that both the trait dimension and item extremity parameters are on a z metric, meaning someone with a trait level of 1.0 is one standard deviation above the mean, and therefore administering that person an item with an extremity parameter of 1.0 would be the exact extremity for which the person has a 50% probability of endorsement. Because items provide maximum information when the probability of endorsement is 50%, it is always most informative to administer items with extremity parameters as close as possible to a person's estimated trait standing. With items calibrated using the 2PLM, they could be used in CAT simulation. Here, 5,000 simulated examinees were administered exactly five items using the catR package in R, where the five items administered to one examinee often differed from those administered to another (39). The frequency with which each item was administered was used to determine the item's quality and suitability for an abbreviated form.

### Qualitative data analysis

The transcription of the focus group was analyzed following the procedures of Miles, Huberman, and Saldana (40), where the research team first reviewed the transcript line by line to identify comments related to the HBM (32). Four of the team members (TC, KF, YL, MF) independently reviewed and coded the transcript using a provisional code list with HBM constructs, then met as a group to review their findings. Codes were revised and the team independently reviewed and recoded the transcript and met four more times to review findings. The codes were revised throughout the process. Discrepancies in coding were resolved through consensus among the first two authors. During second order coding using our final code list, the codes were reviewed to identify semantic relationships in our findings, which were used to group them into themes. Attitudes and knowledge emerged as the two major themes from the analysis.

### Mixed methods integration

We used standard merging and narrative weaving techniques to compare and contrast quantitative and qualitative data (41, 42). Specifically, we examined how changes to the pilot survey resulting from our psychometric analyses did or did not align with recommended changes offered by participants during our qualitative focus groups.

## Results

### Quantitative

Two findings from the psychometric analyses were unexpected. First, the empirical methods strongly suggested a single factor was sufficient to explain inter-item covariance, rather than the two hypothesized factors of “COVID-related” and “HPV-related.” Second, the 2- and 3-factor EFA solutions suggested that, to the extent multiple factors were necessary to explain the covariance, they were better characterized as attitudes/knowledge (Factor 1) and willingness/intention to accept a vaccine (Factor 2). When a third factor was extracted, the additional factor related to knowledge about cancer prevention (cancer_HPV) and intention to seek an HPV vaccine.

Table 2 shows the results of the unidimensional, 2-, and 3-factor solutions of the combined item set. The highest loadings on the unidimensional model were “administer_hpv” (0.95) and “accept_covid” (0.94) indicating that these were the strongest indicators of the measured trait. The lowest loadings were “Intends_No_HPV_Vaccine” (0.21) and “inform_COVID” (0.40), indicating they were the weakest indicators.

**Table 2:** Exploratory factor analysis results on tetrachoric inter-item correlations for the HPV-COVID survey 2.0.

Item	Uni	2-Factor		3-Factor		
		F1	F2	F1	F2	F3
Do you believe that dental professionals are qualified to educate you about the HPV vaccines available? (dental_hpv)	0.93	0.99		0.76	0.31	
Do you believe that dental professionals are qualified to educate you about the COVID-19 vaccines available? (dental_covid)	0.82	0.94		0.57	0.56	
Would you feel comfortable discussing HPV vaccines with your dental providers? (comfort_hpv)	0.90	0.76		0.85		
Would you feel comfortable discussing COVID-19 vaccines with your dental providers? (comfort_covid)	0.91	0.68	0.30	0.67		0.34
Have you been informed that COVID-19 vaccine can prevent severe illness caused by COVID-19? (inform_covid)	0.40	0.68		0.83		
Have you been informed that HPV vaccine can prevent some types of head and nec cancer? (cancer_hpv)	0.52	0.50			0.99	
If not completely vaccinated against HPV, do you plan on receiving the HPV vaccine series? (Intends_No_HPV_Vaccine)	0.21	0.16			0.35	
If your dental provider offered you a HPV vaccine today, would you refuse (Vaccine_Today_No)	0.58	−0.46	1.14	−0.42		1.15
Would you feel comfortable allowing a dental provider to administer a COVID-19 vaccine for you? (administer_covid)	0.93		0.89			0.91
Would you feel comfortable allowing a dental provider to administer an HPV vaccine for you? (administer_hpv)	0.95	0.33	0.71	0.39		0.77
If not completely vaccinated against COVID-19, do you plan on receiving the COVID-19 vaccine? (Intends_No_COVID_Vaccine)	0.73		0.67			0.69
Would you accept the recommendation for an HPV vaccine series from your dental provider? (accept_hpv)	0.92	0.40	0.60	0.35		0.64
Would you accept the recommendation for a COVID-19 from your dental provider? (accept_covid)	0.94	0.42	0.60	0.30		0.62

Loadings < 0.30 removed for clarity unless the dominant loading on the factor is <0.30; rotation = promax; extraction = least-squares; Uni = unidimensional; F = factor.

Table 3 shows the IRT parameters estimates and results of the CAT simulations when the COVID- and HPV-related item sets were used separately as item banks [J] (see Supplementary Table 1 for estimates with confidence intervals). For the COVID items, discrimination parameters were highest for “administer_COVID” (8.15) and “accept_COVID” (9.48), and extremity ranged from −0.21 (“Vaccine_Today_No”) to 5.23 (“inform_COVID”). Note that because items were coded such that endorsement indicated a “no” response, the extreme difficulty of the “inform_COVID” item indicates that almost no one said s/he was *not* informed about COVID. For the HPV items, discrimination parameters were highest for “administer_HPV” (5.53) and “comfort_HPV” (5.51), and extremity ranged from −0.24 (“Vaccine_Today_No”) to 1.80 (“Intends_No_HPV_Vaccine”). For the item administration frequency (quality), the top four COVID-related items were “administer_covid”, “dental_covid”, “Intends_No_COVID_Vaccine”, and “Vaccine_Today_No”. The top four HPV-related items were “accept_hpv”, “administer_hpv”, “Vaccine_Today_No”, and “comfort_hpv.” [J] Notably, variability of these discrimination parameter estimates across items provides support for the use of the 2PL model rather than a 1PL model in which the discrimination parameter is constrained to equality across items.

**Table 3:** Item response theory parameter estimates and frequency of item administration during CAT simulation for COV- and HPV-related items separately.

COVID Item	IRT parameters		CAT sessions administered^a^	Item quality
	*Α*	*β*		
Would you feel comfortable allowing a dental provider to administer a COVID-19 vaccine for you? (administer_covid)	8.15	0.43	5,000	1.00
Do you believe that dental professionals are qualified to educate you about the COVID-19 vaccines available? (dental_covid)	2.24	1.07	4,602	0.92
If not completely vaccinated against COVID-19, do you plan on receiving the COVID-19 vaccine? (Intends_No_COVID_Vaccine)	2.01	0.87	4,217	0.84
If your dental provider offered you a COVID-19 vaccine today, you would like refuse (Vaccine_Today_No)	1.72	−0.21	3,996	0.80
Would you accept the recommendation for a COVID-19 from your dental provider? (accept_covid)	9.48	0.71	1,181	0.24
Would you feel comfortable discussing COVID-19 vaccines with your dental providers? (comfort_covid)	2.72	1.13	1,004	0.20
Have you been informed that COVID-19 vaccine can prevent severe illness caused by COVID-19? (inform_covid)	0.74	5.23	0	0.00
HPV item	IRT parameters		CAT Sessions administered^a^	Item quality
	*Α*	β		
Would you accept the recommendation for an HPV vaccine series from your dental provider? (accept_hpv)	5.48	0.55	5,000	1.00
Would you feel comfortable allowing a dental provider to administer an HPV vaccine for you? (administer_hpv)	5.53	0.40	5,000	1.00
If your dental provider offered you a HPV vaccine today, you would like refuse (Vaccine_Today_No)	1.23	−0.24	3,824	0.76
Would you feel comfortable discussing HPV vaccines with your dental providers? (comfort_hpv)	5.51	0.67	2,998	0.60
Have you been informed that HPV vaccine can prevent some types of head and neck cancer? (cancer_hpv)	0.65	0.54	2,002	0.40
Do you believe that dental professionals are qualified to educate you about the HPV vaccines available? (dental_hpv)	4.91	0.80	1,176	0.24
If not completely vaccinated against HPV, do you plan on receiving the HPV vaccine series? (Intends_No_HPV_Vaccine)	0.23	1.80	0	0.00

^a^ Maximum possible = 5,000; IRT = item response theory; *α* = discrimination parameter; β = difficulty (extremity) parameter; CAT = computerized adaptive testing; HPV = human papillomavirus.

Supplementary Table 2 shows the fit statistics for the COVID and HPV models. The M2 statistic (44) is non-significant, suggesting acceptable fit, and the CFIs/TLIs (all > 0.95) and RMSEAs (both < 0.06) are consistent with this (43, 44). However, the SRMRs (both > 0.08) are higher than usually considered acceptable; this would suggest some highly correlated residuals. Supplementary Table 3 shows the item residual covariances and local dependency statistics, which give some clues as to what is causing the high SRMR. There are indeed some “doublets”, or pairs of items that have significant covariance even after controlling for the latent trait. Examples include dental covid with accept_covid and vaccine_today, admin_hpv with vaccine_today, and cancer_hpv with dental_hpv and int_no_hpv. Notably, the residual covariance between accept_covid and admin_covid was only moderate (0.13), suggesting it is likely not the sole cause of the very high discrimination parameters of those two items.

## Qualitative

### Participant characteristics

Table 4 details demographic characteristics of focus group participants. Of the nine focus group participants, (56%) identified as female. Most participants were 40 years of age or younger (89%), and the mean age of participants was 30 years. Participants identifying as White made up 44% of the focus group (*n* = 4) and participants identifying as Asian made up 44% (*n* = 4).

**Table 4:** Participant characteristics.

Characteristic	*n*
Sex assigned at birth	
Male	4
Female	5
Current gender identity	
Male	4
Female	5
Age	
19–29	4
30–40	4
41–51	1
Race/ethnicity	
White	4
Hispanic/Latinx	0
Black/African American	0
American Indian/Alaska Native	0
Asian	4
Other	1
Education	
Some college	4
Bachelor's degree	3
Master's degree	1
Doctoral/professional degree	1
Household Income	
Less than $20,000	1
$50,001–$75,000	3
$75,001–$100,000	0
Over $100,000	5

### Recommendations for pilot survey changes

Using the think aloud method to get feedback from the participants about the pilot survey, participants noted the following problems: (1) the phrase completely vaccinated is unclear; (2) the answer choices yes, no and unsure did not account for every vaccination status and intention—there should be a prefer not to answer choice; (3) lack of knowledge on the HPV vaccine name; and 5. lack of clarity on what dental professional meant.

As a result of the feedback, changes were made to the pilot survey see Table 5 for details. The term “completely vaccinated” was clearly defined in both the COVID-19 and HPV portion of the final survey. The number of doses required to be considered “up to date” on each type of vaccine was included in the final survey. The pilot survey question “If no, do you plan on receiving the COVID-19 vaccine(s)?” was also combined with the above question and language was added to clarify variations in vaccination status and intention using a single question. A “prefer not to answer” choice was added. The HPV vaccine name, “Gardasil 9” was added. The term “dental professional” was changed to “dentist” for clarification fort the final survey, despite the ability for auxiliary dental personnel to educate and play other roles in vaccination in certain areas.

**Table 5:** Joint display of final survey changes based on psychometrics and focus group findings.

Survey modification	Psychometric finding(s)	Focus group feedback
1. Length of the survey was reduced from 16 questions to 12 (see Supplementary Appendix C). This modification results in a 25% item decrease	Survey items with high discrimination parameters were retained, whereas items with low discrimination demonstrated limited contribution to survey and were dropped (see Table 3)	N/a
2. Demographic questions were moved to the beginning of the survey	Given the strong unidimensional structure of the survey, organizing demographic questions separately from the attitudinal items helped reduce potential context or priming effects and preserve the integrity of the measured construct	N/a
3. COVID vaccine questions now precede HPV vaccine questions and wording for these items are now identical in form	Standardizing wording across vaccine types reduces construct-irrelevant variance and supports comparability when items load on a common factor.	*“Because I think if a large portion of the population doesn't even know what HPV is, like I think a larger portion won't know what the benefits of a vaccine are.”* “*Coronavirus, yes, everyone of all ages right now can understand. But I don't know HPVs are those that everyone has been educated on.”*
4. The term “completely vaccinated” was unclear. The survey now defines this term, including details regarding the number of vaccine doses required to be considered up-to-date on both vaccinations.	Survey item “*Intends_No_HPV_Vaccine*” had very low discrimination and was never administered during CAT sessions. This finding suggests inconsistent interpretation of vaccination status and intent, supporting the need to explicitly define “completely vaccinated,” including dose requirements.	*“But I think we could have a good explanation just to make sure people are more aware when they are taking the survey.”* “*I would also think of does it mean two because that's what we are kind of been conditioned to understand as ‘completely.”*
5. The HPV vaccine name, “Gardasil 9,” was added for clarity.	Knowledge-related HPV items demonstrated lower discrimination (see Table 3). Adding the specific vaccine name was intended to address this ambiguity and improve item interpretability.	*“People may not know what it is. They might need you to give what the vaccine is Gardasil as well?”* “*Like I was younger to get my booster shots at the dentist, they would say Gardasil.”*
6. The term “dental professional” was changed to “dentist” throughout the survey.	“Dental_hpv” item regarding “dental professionals” demonstrated low discrimination (see Table 3)	*“Specifically, have it say dentist, or something more specified. Because dental professional is quite a broad term and I don't think the common person who is not immersed in it would understand the difference.”* “*I would go to the dentist, or when I do go to the dentist, I'll hear, I know both their opinions should be weighted equally because they are both dental professionals, but for me it's kind of like okay the dentist is coming in now, let's see what he has to say.”*
7. “Prefer not to answer” and free-text answer choices were added to the survey.	N/a	*“Like I would venture to guess that my parents have zero clue and they would be like no I am not getting this series I don't even know what you are talking about.”* “*That could be another controversial question for some people. I guess* “*I am not sure*” *is your option for that. I don't know how to rephrase that though.* “*Unsure*” *may be an option.”*

### Themes

The two overarching themes to better understand perceptions of dentist involvement in vaccine efforts identified from the focus group were: (1) attitudes related to appropriateness and acceptability of dental providers educating and administering vaccinations; and (2) how lack of knowledge hindered intentions to seek vaccination from dental providers.

#### Attitudes related to appropriateness and acceptability of dental providers educating and administering vaccinations

Overall, participants shared attitudes that suggested it may not be appropriate or acceptable for dental providers to provide vaccinations. They recognized the need for the dentist to administer COVID-19 vaccinations during the COVID-19 pandemic. They respected the expertise that dentists bring to their profession but would generally question the role of the dentist inquiring about HPV or other vaccinations. They indicated that dentists would not be “staying in their lanes” when discussing or administering vaccinations. For example, one focus group participant stated that “I can see people being like why is my dentist talking to me about the rest of my health and not just my teeth.” While another participant explained, “..bringing up that vaccine can just be quite confusing.”

#### Lack of knowledge hindered intentions to seek vaccination from dental providers

Overall, participants had limited knowledge about the role of dentists educating and providing vaccinations. Participants shared there is not much evidence about the role of dentists publicized, which would make them cautious if a dental provider approached them, stating that “I might if anything go back to my primary care physician and be like “hey, you know what the dentist told me?”” However, they also shared that a conversation by the dentist might have them think more about their role in vaccination. For example, one participant stated that “even if it's just like a brief little mention by the dentists, now [it's in] my mind and it's something to look into,” while another added “I haven't been informed of that. Showing the difference with that HPV vaccine can be helpful. Might be like “oh, I wasn't gonna take it.” Now that I've learned that. It's like, oh, well, it has broader health benefits than maybe I thought.”

### Final survey

Based on the integrated results of the psychometric and focus group analysis, the pilot survey was adjusted. See Table 5 for a joint display of final survey changes based on psychometrics and focus group findings. Items were considered for inclusion and edited based on a combination of qualitative and quantitative metrics, and the goal of the survey. The length of the final survey was organized according to factor analysis and modified to reflect the recommendations provided by focus group participants (see Supplementary Appendix C). These changes reduced the survey from 22 questions to 16 questions, a 25% decrease. The final survey included one question about both actual behavior and intention, one question about knowledge, and three questions about attitudes, compared to one question each about actual behavior, intention, and knowledge in the original survey and 5 questions about attitudes. Patients were also provided free-text space to provide comments at the end of the COVID-19 and HPV vaccine questions. A version history table that reconciles the 1. original pilot items and response options; 2. The exact item set used for the EFA/IRT/CAT; and 3. The final 16 item instrument are presented in Table 6.

**Table 6:** Version history table.

Survey items	Original pilot items	Item set for EFA/IRT/CAT	Final 16 item
What sex were you assigned at birth? (Male Female)	x		x
What is your current gender identity (please select all that apply)? (Male, Female, Transgender, Non-binary, Gender variant/Nonconforming, Other, Other gender identify (please specify)	x		x
Ages 18–45 (drop-down menu)	x		x
Please specify your race/ethnicity (check all that apply) (White, Hispanic/Latinx, Black/African American, American Indian/Alaska Native, Asian, Native Hawaiian/Other Pacific Islander, Prefer not to say, Other (please specify)	x		x
What is the highest level of education that you have completed? (less than high school, high school diploma or equivalent, some college, bachelor's degree, master's degree, doctorate/ professional degree	x		x
Which of the following describes your household income? (less than $20,000, $20,001-$35,000, $35,001-$50,000, $50,001 - $75,000, $75,001-$100,000, over $100,000	x		x
Have you been completely vaccinated against COVID-19 (Yes, No, Unsure)	x	x	
If no, do you plan on receiving the COVID-19 vaccination(s) (Yes, No, Unsure)	x	x	
Have you been informed that the COVID-19 vaccine can prevent severe illness caused by COVID-19? (Yes, No, Unsure)	x	x	
Do you believe that dental professionals are qualified to educate you about the COVID-19 vaccines available? (Yes, No, Unsure)	x	x	x*
Would you feel comfortable discussing COVID-19 vaccines with your dental providers? (Yes, No, Unsure)	x	x	x*
Would you accept the recommendation for a COVID-19 vaccine from your dental provider? (Yes, No, Unsure)	x	x	
Would you feel comfortable allowing a dental provider to administer a COVID-19 for you? (Yes, No, Unsure)	x	x	x*
If your dental provider offered you a COVID-19 vaccine today, you would likely (accept the vaccine, refuse the vaccine, not applicable (already vaccinated or awaiting a scheduled vaccination appointment)	x	x	
Have you been completely vaccinated against human papillomavirus (HPV)? (Yes, No, Unsure)	x	x	
If no, do you plan on receiving the human papillomavirus (HPV) vaccine series? (Yes, No, Unsure)	x	x	
Have you been informed that the human papillomavirus (HPV) vaccine can prevent some types of head and neck cancer? (Yes, No, Unsure)	x	x	
Do you believe that dental professionals are qualified to educate you about the human papillomavirus (HPV) vaccines available? (Yes, No, Unsure)	x	x	x*
Would you feel comfortable discussing human papillomavirus (HPV) vaccines with your dental providers? (Yes, No, Unsure)	x	x	x*
Would you accept the recommendation for a human papillomavirus (HPV) vaccine series from your dental provider? (Yes, No, Unsure)	x	x	
Would you feel comfortable allowing a dental provider to administer an human papillomavirus (HPV) vaccine for you? (Yes, No, Unsure)	x	x	x*
If your dental provider offered you an human papillomavirus (HPV) vaccine today, you would likely (accept the vaccine, refuse the vaccine, not applicable (already vaccinated or awaiting a scheduled vaccination appointment)	x	x	
Are you, or do you plan to become, up to date on your COVID-19 vaccinations? This could include the 1 dose of the Johnson and Johnson vaccination or the 2 doses of the Moderna or Pfizer vaccinations, plus any boosters applicable to you (Yes, I have received ALL recommended vaccination does, yes, I have received SOME of the vaccination does and I am planning to receive them all in the future, No, I have NOT received any of the vaccination doses, but I AM planning to receive them in the future, No, I have NOT received any of the vaccination does and am NOT planning to receive any of them in the future, Unsure, Prefer not to answer)			x
Have you been informed before today that the COVID-19 vaccine can prevent severe illness caused by COVID-19? (Yes, No, Unsure)			x
Are you, or do you plan to become, up to date on your human papillomavirus (HPV) vaccination? This could include 2 doses of the Gardasil 9 vaccination if received before 15 years of age or 3 doses if received at or after 15 years. (Yes, I have received ALL recommended vaccination does, yes, I have received SOME of the vaccination does and I am planning to receive them all in the future, No, I have NOT received any of the vaccination doses, but I AM planning to receive them in the future, No, I have NOT received any of the vaccination does and am NOT planning to receive any of them in the future, Unsure, Prefer not to answer)			x
Have you been informed before today that the human papillomavirus (HPV) vaccine can prevent some types of head and neck cancer? (Yes, No, Unsure)			x

## Discussion

Our study used a mixed methods approach to assess the initial validity of a new survey and to understand perceptions of dentist involvement in vaccine administration. Using integrated quantitative and qualitative methodology, the resultant survey is an initial psychometrically valid instrument supported by feedback from the community regarding clarity and accessibility of the questions. The removal and rewording of redundant items underscore the importance of both quantitative and qualitative feedback in creating an effective and reliable survey tool. The Item Response Theory (IRT) analyses revealed that certain items were low-quality, high-difficulty, and zero-administration in CAT, which led to removal or rewording.

The high discrimination observed in HPV-related items underscores their key role in assessing patient attitudes toward dental providers administering HPV vaccines, indicating the necessity for focused communication strategies. For COVID-19, items such as “administer_covid” and “accept_covid” had very high discrimination parameters (*α*), indicating their strong ability to differentiate between respondents with varying attitudes towards COVID-19 vaccination by dental providers. These items were frequently administered in CAT sessions highlighting their importance and informativeness. In contrast, “inform_covid” (*α* = 0.74, *β* = 5.23) showed low discrimination and was not administered in any CAT session, reflecting its limited contribution to the survey's effectiveness.

For HPV, items like “accept_hpv” and “administer_hpv” also demonstrated high discrimination and were administered frequently, indicating their critical role in capturing patient attitudes towards HPV vaccination by dental providers. However, “Intends_No_HPV_Vaccine” (*α* = 0.23, *β* = 1.80) had very low discrimination and was never administered during CAT sessions, suggesting it may not be useful for the final survey.

By addressing both the attitudes towards information received from dental providers and the willingness to accept vaccines, along with the knowledge about HPV, the survey can provide comprehensive insights that can inform targeted interventions and communication strategies.

The factor analysis revealed that a single factor, such as high-loading items, “administer_hpv” and “accept_covid” provide a clear picture of patient perceptions and also play a crucial role in understanding the role of dental providers in vaccination. Targeted interventions can be guided based on those items. The 3-factor solution, represented by items such as “cancer_hpv” and “Intends_No_HPV_Vaccine” indicates the crucial role of knowledge in shaping patients’ attitudes and intentions towards receiving the HPV vaccine from dental providers. This suggests that educational interventions focusing on HPV-related information could significantly influence patient attitudes and acceptance of vaccination from dental providers.

These results were supported by the comments of the focus group participants who noted redundancies, the need for rewording, and their overall attitudes related to the appropriateness and acceptability of dental providers educating and administering vaccinations. Their comments also showed that their lack of knowledge hindered intentions to seek vaccination from dental providers.

It is important to note that despite the higher levels of education of participants, the focus group suggested several changes to make the survey more understandable. Using qualitative methods such as focus groups with the targeted community ensures that participants clearly understand what the questions are asking—in this way, researchers can be sure that they are getting responses to questions they intend to get.

Lack of knowledge seemed to be the basis for not understanding the role of the dental provider and not understanding the link between HPV and oropharyngeal squamous cell carcinomas (45, 46). This lack of knowledge affected attitudes about dental involvement in vaccination programs. To address the knowledge gaps, clear and understandable language is needed when communicating with patients. Ensuring that patients fully understand the connection and the importance of dental providers in this context can enhance their acceptance and support of vaccination initiatives.

Consistent with other studies, participants were mixed in their attitudes about dental providers administering vaccinations, questioning why this was needed and if their qualifications were appropriate (1, 47). While they understood that this may have been necessary during the COVID-19 pandemic given demand, they were less clear about the role of dental providers administering vaccinations during times of less demand. Further, they seemed to feel more strongly about dental providers not administering the HPV vaccination than they did about dental providers administering the COVID-19 vaccine. This may be due to a lack of knowledge about the HPV vaccination, which was a theme from the focus group analysis. However, it is important to note that previous studies suggest parents support dental providers administering HPV vaccines to their children and discussing oral cancer with them, which is in contrast to these perspectives of adult patients (14). Further, most states do not allow dentists to administer vaccines and according to some studies (48, 49), slightly more than half (55%) of dentists are willing to provide vaccines to patients. Thus, states that do not endorse dentists to provide vaccinations and dentists who do not discuss vaccination options could be a potential reason why participants may wonder why dentists are talking about more than their teeth.

Participants’ comments provide formative data to develop educational interventions that address the lack of knowledge about what HPV vaccines are, the connection to oral health, and the roles dentists can play in their overall health. Testing educational interventions with patients can improve shared decision-making, patient-centered care, and patient knowledge about the crucial role of dental providers and vaccination programs.

The study limitations include conducting one focus group in an urban setting. While the aim was not to generalize the findings from the focus group, multiple focus groups with varying levels of participant education could have elicited other recommendations for survey changes. This limitation is balanced by the study's initial psychometric analysis. Future research should test the survey with participants with lower educational status or lower health literacy given that the average American adult has an 8th grade level of education, or below (50). A second limitation is that the assumptions of IRT modeling were not fully met; the local independence assumption was violated for some pairs of items. The acceptable model fit across all indices except the SRMR supports the unidimensionality assumption, where any indications of multidimensionality are likely due to local correlated residuals; however, the violations of local independence could cause some discrimination parameter estimates to be inflated (thus inflating information estimates). Future validation of this scale will help answer the question of whether inflated information estimates in the CAT simulation procedure in this study led to premature stopping (and therefore “over-shortening” of the scale). Finally, it is important to note that the sample size used here is below most recommended cutoffs/minima for IRT modeling (38), which means future investigating of this question with a larger sample is highly advisable.

### Public health implications

Given the number of people in the US that visit their dental providers more (47, 51) than their physicians, and the ongoing work needed to increase vaccination coverage, we need valid surveys that assess attitudes related to dentist roles in preventive healthcare. Testing surveys with target populations provides a crucial opportunity to engage the population and understand their perceptions to develop interventions. If findings from future research using this survey indicate that patient acceptance is high for dentists to provide vaccinations, there are opportunities to educate legislators and advocate for changes in what professions are allowed to vaccinate. Expanding the scope of dentistry to include vaccine education and administration could increase access to care, vaccine uptake, and disease prevention. This study not only provides evidence for a valid survey to test without concerns regarding understandability and clarity but also provides evidence that in our efforts to create a comprehensive public health system. We need all health care providers involved in vaccination efforts. Every clinical encounter is an opportunity to vaccinate (52).

## Data Availability

The datasets presented in this article are not readily available because of privacy concerns. Requests to access the datasets should be directed to the corresponding author.
