# Supplementary material for: Using psychometric and focus groups methods to assess patients' attitudes regarding the role of dental providers and vaccinations for COVID-19 and HPV
Source: Front Oral Health. 2026 Feb 20;7:1740318. doi: 10.3389/froh.2026.1740318 (PMC12963340; doi:10.3389/froh.2026.1740318)
Supplement: Supplementary file 1 [file Table1.docx]

| Table 1a. Fit Indices for 2PL IRT models. | | |
| --- | --- | --- |
| Metric | COVID | HPV |
| M2 (df \| p) | 20.1 (14 \| 0.127) | 19.4 (14 \| 0.150) |
| RMSEA (CI) | 0.052 (0.000 - 0.098) | 0.049 (0.000 - 0.096) |
| SRMR | 0.090 | 0.082 |
| TLI | 0.966 | 0.972 |
| CFI | 0.978 | 0.981 |
| Note. HPV = human papillomavirus; RMSEA = root mean-square error of approximation; CI = 95% confidence interval; SRMR = standardized root mean-square residual; TLI = Tucker-Lewis Index; CFI = Comparative Fit Index. | | |
|  | | |

Supplementary Table 1

| Table 1b. Item Parameter Estimates (and Confidence Intervals) for 2PL Models. | | |
| --- | --- | --- |
| COVID | | |
| Item | a (95% CI) | b (95% CI) |
| inform_covid | 0.739 (-0.495 - 1.973) | 5.23 (-2.558 - 13.018) |
| dental_covid | 2.239 (0.916 - 3.562) | 1.074 (0.622 - 1.527) |
| comfort_covid | 2.716 (1.278 - 4.153) | 1.132 (0.771 - 1.493) |
| accept_covid | 9.485 (-5.789 - 24.758) | 0.711 (0.472 - 0.95) |
| administer_covid | 8.151 (-1.843 - 18.146) | 0.426 (0.207 - 0.645) |
| Intends_No_COVID_Vaccine | 2.011 (1.084 - 2.939) | 0.867 (0.541 - 1.194) |
| Vaccine_Today_REVERSED | 1.716 (0.897 - 2.535) | -0.206 (-0.481 - 0.069) |
| HPV | | |
| Item | a (95% CI) | b (95% CI) |
| cancer_hpv | 0.654 (0.17 - 1.137) | 0.536 (-0.126 - 1.198) |
| dental_hpv | 4.914 (1.21 - 8.617) | 0.801 (0.525 - 1.077) |
| comfort_hpv | 5.514 (1.423 - 9.604) | 0.672 (0.428 - 0.915) |
| accept_hpv | 5.48 (1.674 - 9.286) | 0.55 (0.314 - 0.786) |
| administer_hpv | 5.526 (1.781 - 9.271) | 0.395 (0.163 - 0.628) |
| Intends_No_HPV_Vaccine | 0.228 (-0.174 - 0.631) | 1.805 (-1.591 - 5.2) |
| Vaccine_Today_REVERSED | 1.23 (0.605 - 1.856) | -0.245 (-0.578 - 0.089) |
| Note. CI = confidence interval; HPV = human papillomavirus. | | |

| Table 1c. Local Dependency Statistics for 2PL IRT Models | | | | | | | |
| --- | --- | --- | --- | --- | --- | --- | --- |
| COVID | | | | | | | |
| Item | inform_covid | dental_covid | comfort_covid | accept_covid | admin_covid | Int_No_COVID | Vaccine_Today |
| inform_covid |  | 0.115 | 0.083 | 0.141 | -0.064 | 0.065 | -0.090 |
| dental_covid | 1.565 |  | 0.195 | 0.251 | 0.178 | -0.133 | -0.256 |
| comfort_covid | 1.013 | 4.423 |  | -0.134 | -0.061 | 0.087 | -0.092 |
| accept_covid | 2.670 | 6.842 | 2.394 |  | 0.130 | 0.190 | -0.158 |
| admin_covid | 0.521 | 3.375 | 0.470 | 2.000 |  | 0.051 | 0.158 |
| Int_No_COVID | 0.673 | 2.137 | 1.142 | 4.938 | 0.350 |  | 0.017 |
| Vaccine_Today | 1.265 | 7.853 | 1.278 | 3.403 | 3.338 | 0.050 |  |
| HPV | | | | | | | |
| Item | cancer_hpv | dental_hpv | comfort_hpv | accept_hpv | admin_hpv | Int_No_HPV | Vaccine_Today |
| cancer_hpv |  | 0.215 | 0.030 | 0.080 | 0.123 | 0.202 | -0.037 |
| dental_hpv | 4.667 |  | 0.120 | 0.048 | -0.126 | 0.095 | -0.133 |
| comfort_hpv | 0.112 | 1.501 |  | -0.069 | 0.051 | 0.044 | -0.106 |
| accept_hpv | 0.744 | 0.226 | 0.588 |  | 0.094 | 0.091 | 0.112 |
| admin_hpv | 1.704 | 1.483 | 0.303 | 1.025 |  | 0.112 | 0.225 |
| Int_No_HPV | 5.605 | 0.960 | 0.257 | 1.060 | 1.547 |  | 0.052 |
| Vaccine_Today | 0.186 | 1.921 | 1.515 | 1.615 | 6.334 | 0.432 |  |
| Note. Local dependency statistics below diagonal; Cramer's V above diagonal; HPV = human papillomavirus; Int = intends; admin = administration. | | | | | | | |
