# Supplementary material for: Using psychometric and focus groups methods to assess patients' attitudes regarding the role of dental providers and vaccinations for COVID-19 and HPV
Source: Front Oral Health. 2026 Feb 20;7:1740318. doi: 10.3389/froh.2026.1740318 (PMC12963340; doi:10.3389/froh.2026.1740318)
Supplement: Supplementary file 2 [file Table2.docx]

Table 2: Appendices

Appendix A

*Demographics*

1. What sex were you assigned at birth?
   - Male
   - Female

1. What is your current gender identity (please select all that apply)?

- Male
- Female
- Transgender
- Non-binary
- Gender variant/nonconforming
- Other
- Other gender identity (please specify) ______

1. Age: 18 - 45 (drop down menu)
2. Please specify your race/ethnicity (check all that apply)?
   - White
   - Hispanic/Latinx
   - Black/African American
   - American Indian/Alaska Native
   - Asian
   - Native Hawaiian/Other Pacific Islander
   - Prefer not to say
   - Other (please specify) ________
3. What is the highest level of education that you have completed?
   - Less than high school
   - High school diploma or equivalent
   - Some college
   - Bachelor’s degree
   - Master’s degree
   - Doctorate/professional degree
4. Which of the following best describes your household income?
   - Less than $20,000
   - $20,001 to $35,000
   - $35,001 to $50,000
   - $50,001 to $75,000
   - $75,001 to $100,000
   - Over $100,000

*Survey Questions*

*COVID-19*

1. Have you been completely vaccinated against COVID-19?

- - Yes
  - No
  - Unsure

2. If no, do you plan on receiving the COVID-19 vaccine(s)?

- - Yes
  - No
  - Unsure

3. Have you been informed that the COVID-19 vaccine can prevent severe illness caused by COVID-19? (inform_covid)

- - Yes
  - No
  - Unsure

4. Do you believe that dental professionals are qualified to educate you about the COVID-19 vaccines available? (dental_covid)

- - Yes
  - No
  - Unsure

5. Would you feel comfortable discussing COVID-19 vaccines with your dental providers? (comfort_covid)

- - Yes
  - No
  - Unsure

6. Would you accept the recommendation for a COVID-19 vaccine from your dental provider? (accept_covid)

- - Yes
  - No
  - Unsure

7. Would you feel comfortable allowing a dental provider to administer a COVID-19 for you? (administer_covid)

- - Yes
  - No
  - Unsure

8. If your dental provider offered you a COVID-19 vaccine today, you would likely: (vaccine_today)

- - Accept the vaccine
  - Refuse the vaccine
  - Not applicable (i.e. already vaccinated or awaiting a scheduled vaccination appointment)

9. Please provide any additional comments on your attitudes towards dental providers’ role in administering the COVID-19 vaccine:

*Human Papillomavirus (HPV)*

1. Have you been completely vaccinated against human papillomavirus (HPV)?

- - Yes
  - No
  - Unsure

2. If no, do you plan on receiving the human papillomavirus (HPV) vaccine series?

- - Yes
  - No
  - Unsure

3. Have you been informed that the human papillomavirus (HPV) vaccine can prevent some types of head and neck cancer? (cancer_hpv)

- - Yes
  - No
  - Unsure

4. Do you believe that dental professionals are qualified to educate you about the human papillomavirus (HPV) vaccines available? (dental_hpv)

- - Yes
  - No
  - Unsure

5. Would you feel comfortable discussing human papillomavirus (HPV) vaccines with your dental providers? (comfort_hpv)

- - Yes
  - No
  - Unsure

6. Would you accept the recommendation for a human papillomavirus (HPV) vaccine series from your dental provider? (accept_HPV)

- - Yes
  - No
  - Unsure

7. Would you feel comfortable allowing a dental provider to administer an human papillomavirus (HPV) vaccine for you? (administer_hpv)

- - Yes
  - No
  - Unsure

8. If your dental provider offered you an human papillomavirus (HPV) vaccine today, you would likely: (Vaccine_Today)

- - Accept the vaccine
  - Refuse the vaccine
  - Not applicable (i.e. already vaccinated or awaiting a scheduled vaccination appointment)

9. Please provide any additional comments on your attitudes towards dental providers’ role in administering human papillomavirus (HPV) vaccines:

Appendix B

Focus Group Guide

Using the “think-aloud” method,^1^ we will review our survey with participants. While we are interested in participants’ responses, we are particularly interested in their feedback and their thoughts about the survey questions.

1. **Welcome participants and thank them for coming**

*Thank you for joining our focus group. For the next 60 minutes, we will be having a discussion to understand your thoughts and feelings about the role of dental providers and your care. As individuals who come to the dental clinic, you are the experts. We want to know the best way to make your care better. Now, we will introduce ourselves. Are there any questions before we get started?*

*Before we begin our discussion, we want to go over a few brief points:*

*During the discussion ________Names of co-moderators) will be listening taking notes. They are taking notes so that when the group is over, we will write about the things you said. As we mentioned in our initial contacts with each of you, this conversation is being audio recorded. Your answers are really important to us and so we would like to record them to make sure we don’t miss anything. We plan to share a summary of what we have learned from you with other members of our research team, to revise a survey that will be given to other patients. Eventually, we hope to share or findings with other people doing this type of research, possible through scientific journal articles or even newsletters. Please know that your identity will be protected at all times. We know that you may have both positive and negative things to share and it is important for us to know what you really think. There are no right or wrong answers; we are just trying to learn from you. We really want to better understand what you think about each of the topics that we are about to discuss. It is also important say that no matter what you say, no one will be negatively affected in any way. Hopefully, we will be able to make some important improvements as we learn from your suggestions and feedback. Are there any questions?*

*Have any of you participated in, or conducted focus groups in the past? (acknowledge participants previous experiences in Focus groups.) In these focus groups we have some basic ground rules. In order to be able to hear what each person has to say, please give your attention to the person who is speaking and please avoid having side conversations. It is very important to have one conversation at a time during the focus group because we think that everything that you have to say is important. In order to respect each other’s privacy, we ask that you consider all of the information that is shared here as confidential. Please do not talk about this or share the specifics of this discussion with other people after you leave this room.*

*Again, we really want to know what each of you think. There are no right or wrong answers to any of the questions we will ask you. You all may have a lot of different ideas and experiences, and we want to hear about them. Make sure that you stop us if we say something that doesn’t make sense or if you have questions about anything.*

**2. Make introductions.**

*We’d like to get started by having everyone introduce themselves. The name on your name tag should be what you would like us to call you.*

1. **Provide the purpose of the study and explain how the focus group fits into the project as a whole**
2. **Begin focus group activities.**

I will be asking a series of questions and we are interested in your thoughts and responses; remember there are no right or wrong answers. *We will be asking the survey questions only, not the demographics*. We will probe when necessary. For example, if a participant has a question about one of the survey questions, we will probe about why the question exists in addition to answering it.

1. Give us your thoughts and feedback about the following:
   1. Have you been completely vaccinated against COVID-19?
   2. If no, do you plan on receiving the COVID-19 vaccine?
   3. Have you been informed that the COVID-19 vaccine can prevent severe illness caused by COVID-19?
   4. Do you believe that dental professionals are qualified to educate you about the COVID-19 vaccines available?
   5. Would you feel comfortable discussing COVID-19 vaccines with your dental provider?
   6. Have you been completely vaccinated against human papillomavirus (HPV)?
   7. If no, do you plan on receiving the human papillomavirus (HPV) vaccine series?
   8. Have you been informed that the human papillomavirus (HPV) vaccine can prevent some types of head and neck cancer?
   9. Do you believe that dental professionals are qualified to educate you about the human papillomavirus (HPV) vaccines available?
   10. Would you feel comfortable discussing human papillomavirus (HPV) vaccines with your dental provider?
2. **End the meeting.**

*Thank you for sharing your thoughts and experiences with us. Your knowledge and insights are a crucial part of our research. If you have any questions or concerns, please do not hesitate to contact us.*

Appendix C

*Demographics*

1. What sex were you assigned at birth?
   - Male
   - Female

1. What is your current gender identity (please select all that apply)?

- Male
- Female
- Transgender
- Non-binary
- Gender variant/nonconforming
- Other
- Other gender identity (please specify) ______

1. Age: 18 - 45 (drop down menu)
2. Please specify your race/ethnicity (check all that apply)?
   - White
   - Hispanic/Latinx
   - Black/African American
   - American Indian/Alaska Native
   - Asian
   - Native Hawaiian/Other Pacific Islander
   - Prefer not to say
   - Other (please specify) ________
3. What is the highest level of education that you have completed?
   - Less than high school
   - High school diploma or equivalent
   - Some college
   - Bachelor’s degree
   - Master’s degree
   - Doctorate/professional degree
4. Which of the following best describes your household income?
   - Less than $20,000
   - $20,001 to $35,000
   - $35,001 to $50,000
   - $50,001 to $75,000
   - $75,001 to $100,000
   - Over $100,000

*Survey Questions*

*COVID-19*

1. Are you, or do you plan to become, up to date on your COVID-19 vaccinations? This could include the 1 dose of the Johnson and Johnson vaccination or the 2 doses of the Moderna or Pfizer vaccinations, plus any boosters applicable to you.

- - Yes, I have received ALL recommended vaccination doses
  - Yes, I have received SOME of the vaccination doses and I am planning to receive them all in the future
  - No, I have NOT received any of the vaccination doses, but I AM planning to receive them in the future
  - No, I have NOT received any of the vaccination doses and am NOT planning to receive any of them in the future
  - Unsure
  - Prefer not to answer

2. Have you been informed before today that the COVID-19 vaccine can prevent severe illness caused by COVID-19? (inform_covid)

- - Yes
  - No
  - Unsure

3. Do you believe that dentists are qualified to educate you about the COVID-19 vaccines available? (dental_covid)

- - Yes
  - No
  - Unsure

4. Would you feel comfortable discussing COVID-19 vaccines with your dentist? (comfort_covid)

- - Yes
  - No
  - Unsure

5. Would you feel comfortable allowing a dentist to administer a COVID-19 for you (assuming they received the proper training)? (administer_covid)

- - Yes
  - No
  - Unsure

6. Please provide any additional comments on your attitudes towards dental providers’ role in administering the COVID-19 vaccine:

*Human Papillomavirus (HPV)*

1. Are you, or do you plan to become, up to date on your human papillomavirus (HPV) vaccination? This could include 2 doses of the Gardasil 9 vaccination if received before 15 years of age or 3 doses if received at or after 15 years.

- - Yes, I have received ALL recommended vaccination doses
  - Yes, I have received SOME of the vaccination doses and I am planning to receive them all in the future
  - No, I have NOT received any of the vaccination doses, but I AM planning to receive them in the future
  - No, I have NOT received any of the vaccination doses and am NOT planning to receive any of them in the future
  - Unsure
  - Prefer not to answer

2. Have you been informed before today that the human papillomavirus (HPV) vaccine can prevent some types of head and neck cancer? (cancer_hpv)

- - Yes
  - No
  - Unsure

3. Do you believe that dentists are qualified to educate you about the human papillomavirus (HPV) vaccines available? (dental_hpv)

- - Yes
  - No
  - Unsure

4. Would you feel comfortable discussing human papillomavirus (HPV) vaccines with your dentist? (comfort_hpv)

- - Yes
  - No
  - Unsure

5. Would you feel comfortable allowing a dentist to administer a human papillomavirus (HPV) vaccine for you (assuming they received the proper training)? (administer_hpv)

- - Yes
  - No
  - Unsure

6. Please provide any additional comments on your attitudes towards dental providers’ role in administering human papillomavirus (HPV) vaccines:
